# Shared genetic risk between anorexia nervosa and cardiovascular disease events: Evidence from genome‐wide association studies

**DOI:** 10.1002/brb3.3294

**Published:** 2024-01-28

**Authors:** Baiyu Qi, Mariaelisa Graff, Eating Disorders Working Group of the Psychiatric Genomics Consortium, Cynthia M Bulik, Kari E North, Melissa A Munn‐Chernoff

**Affiliations:** ^1^ Department of Epidemiology University of North Carolina at Chapel Hill Chapel Hill North Carolina US; ^2^ Department of Psychiatry University of North Carolina at Chapel Hill Chapel Hill North Carolina US; ^3^ Department of Medical Epidemiology and Biostatistics Karolinska Institutet Stockholm Sweden; ^4^ Department of Nutrition University of North Carolina at Chapel Hill Chapel Hill North Carolina US; ^5^ Department of Community, Family, and Addiction Sciences Texas Tech University Lubbock Texas US

**Keywords:** genetics, heart disease, medical complications

## Abstract

**Objective:**

Cardiovascular complications occur in up to 80% of patients with anorexia nervosa (AN), yet the underlying mechanisms warrant further investigation. We assessed the genetic correlation (*r_g_*) between AN and cardiovascular disease (CVD) events to inform whether elevated cardiovascular risk among individuals with AN is due to shared genetic effects.

**Method:**

We used genome‐wide association study summary statistics for AN (*N* = 72,517), AN with binge eating (*N* = 12,630), AN without binge eating (*N* = 12,516), and six CVD events (*N* = 390,142 to 977,323). We calculated the *r_g_* s via linkage disequilibrium score regression and corrected for multiple testing using false discovery rate.

**Results:**

Significant *r_g_* s emerged between AN with heart failure (*r_g_* = –0.11, SE = 0.05, *q* = .04) and myocardial infarction (*r_g_* = –0.10, SE = 0.03, *q* = .01). AN with binge eating had a significant *r_g_* with myocardial infarction (*r_g_* = –0.15, SE = 0.06, *q* = .02). No significant *r_g_* emerged between AN without binge eating and any CVD event.

**Discussion:**

Some loci affect the liability to AN and CVD in opposite directions and the shared genetic effects may not be consistent across all CVD events. Our results provide further evidence suggesting that the elevated cardiovascular risk in AN may not be due to shared genetic underpinnings, but more likely a downstream consequence of the disease.

## Graphical abstract

We calculated the genetic correlation between three anorexia nervosa (AN) phenotypes and six cardiovascular disease (CVD) events. We observed significant (q<.05) genetic correlations between AN with heart failure and myocardial infarction, and between AN with binge eating and myocardial infarction. No significant genetic correlation emerged between AN without binge eating and any CVD event.

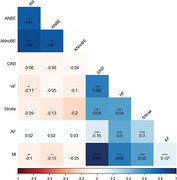

## 1 INTRODUCTION

Cardiovascular complications occur in up to 80% of patients with anorexia nervosa (AN) and are considered the causal substrate for the poor prognosis of AN, accounting for 30% of the mortality in AN (Di Cola et al., 2014; Giovinazzo et al., 2019; Mehler et al., 2022; Spaulding‐Barclay et al., 2016). Common cardiovascular complications observed in patients with AN include structural complications (e.g., pericardial effusion), conduction complications (e.g., QT‐interval prolongation), and hemodynamic changes (e.g., bradycardia) (Giovinazzo et al., 2019; Sachs et al., 2016). Many of these complications are thought to be a result from chronic starvation, low body weight, and malnutrition, yet cardiovascular risks in AN are multifaced and may involve multiple pathways, such as through the neuroendocrine, immune, and autonomic nervous systems (Sachs et al., 2016; Sekaninova et al., 2020). Importantly, a consensus on management practices for the screening and monitoring of cardiac events in AN is still lacking. A comprehensive understanding of pathways contributing to higher risk of cardiovascular diseases (CVD) in AN is critical for screening and monitoring for cardiac events in AN, as well as developing prevention and treatment strategies for elevated cardiac morbidity and mortality observed in AN.

In most cases, cardiac complications of AN are assumed to be sequelae of starvation or electrolyte imbalances. However, shared genetic contributions to AN and CVD have not yet been explored as an alternative or supplementary explanation. Genetic susceptibility underlies AN and most CVD and their antecedents (Aragam et al., 2018; Duncan et al., 2017; Watson et al., 2019). Genome‐wide association studies (GWAS) for AN have demonstrated initial evidence of shared genetic risks between AN and some CVD risk factors: AN had a positive genetic correlation with high‐density lipoprotein cholesterol (HDL‐C) and negative genetic correlations with insulin resistance and type‐2 diabetes, suggesting that genetic factors that increase risk for AN may also increase risk for high HDL‐C and decrease risk for insulin resistance and type‐2 diabetes (Duncan et al., 2017; Watson et al., 2019). However, no study has examined shared genetic risks between AN and CVD events. Moreover, no study has examined differences in CVD risks by AN subtype (e.g., it is unclear whether shared genetic risks between AN and CVD differ between individuals with AN who do versus do not engage in recurrent binge‐eating episodes as part of their symptom profile of their illness). Phenotypically, the behavior of binge eating and threshold binge‐eating disorder have been associated with elevated CVD risk factors such as hypertension and dyslipidemia (Hudson et al., 2010; Mitchell et al., 2015; Thornton et al., 2017). Using polygenic risk scores (i.e., the weighted sum of common risk variants per individual), positive associations have been reported between binge‐eating disorder and CVD risk factors such as type‐2 diabetes and fasting insulin (Hübel et al., 2021). Taken together, these findings indicated that some loci affect AN and CVD risks in the opposite direction, whereas some loci affect binge eating and CVD risks in the same direction, suggesting distinct etiologies underlying CVD risk in AN compared with binge eating. Accordingly, it is important to isolate the contribution of binge eating to any observed shared genetic risk between AN and CVD.

We examined genetic correlations between three AN phenotypes (i.e., AN, AN with binge eating, and AN without binge eating) with six CVD events. We hypothesized that (1) negative genetic correlations would emerge between AN phenotypes with CVD events given previously observed negative genetic correlations between AN with CVD risk factors (Duncan et al., 2017; Watson et al., 2019) and (2) the magnitude of the genetic correlations would be larger for AN without binge eating compared with AN with binge eating. Findings from this study could further elucidate the underlying biology of elevated risk of CVD in patients with AN.

## 2 METHODS

### 2.1 Participants

Samples included in this study are described in Table 1. We used published summary statistics from the Eating Disorders Working Group of the Psychiatric Genomics Consortium (PGC‐ED) for AN (16,992 cases, 55,525 controls), AN with binge eating (2381 cases, 10,249 controls), and AN without binge eating (2262 cases, 10,254 controls) (Watson et al., 2019). We also used published summary statistics for six CVD events, including coronary artery disease (CAD; 122,733 cases, 424,528 controls; van der Harst & Verweij, 2018), heart failure (47,309 cases, 930,014 controls; Shah et al., 2020), nonischemic cardiomyopathy (NICM; 1816 cases, 388,326 controls; Aragam et al., 2018), stroke (67,162 cases, 454,450 controls; Malik et al., 2018), atrial fibrillation (65,446 cases, 522,744 controls; Roselli et al., 2018), and myocardial infarction (61,000 cases, 577,716 controls; Hartiala et al., 2021). All samples were primarily of European ancestry, with less than 10% of samples being ancestrally diverse.

**TABLE 1:** Definition and data for phenotypes included in analyses.

Study	Sample/consortium	Phenotype	Definition	*N* (cases/controls)
Watson et al. (2019)	PGC‐ED	AN	DSM‐III‐R, DSM‐IV, ICD‐8, ICD‐9, ICD‐10, or self‐reported AN	16,992/55,525
		AN with BE		2381/10,249
		AN without BE		2262/10,254
van der Harst and Verweij (2018)	UK BioBank, CARDIoGRAMplusC4D	CAD	ICD‐10 or self‐reported CAD	122,733/424,528
Shah et al. (2020)	HERMES	HF	Physician diagnosis or adjudication, ICD codes, imaging, natriuretic peptides, or self‐reported treatment for HF	47,309/930,014
Aragam et al. (2018)	UK BioBank	NICM	ICD‐9 or ICD‐10	1816/388,326
Malik et al. (2018)	MEGASTROKE	Stroke	Physician diagnosis or adjudication, ICD codes, imaging, or self‐report stroke	67,162/454,450
Roselli et al. (2018)	AFGen, Broad AF, etc.	AF	Physician diagnosis or adjudication, ICD codes, imaging	65,446/522,744
Hartiala et al. (2021)	UK BioBank, CARDIoGRAMplusC4D	MI	ICD10, doctor‐diagnoses, or self‐reported MI	∼61,000/577,716

AF: atrial fibrillation; AFGen: Atrial Fibrillation Genetics consortium; AN: anorexia nervosa; BE: binge eating; CAD: coronary artery disease; CARDIoGRAMplusC4D: Coronary ARtery DIsease Genome‐wide Replication and Meta‐analysis plus The Coronary Artery Disease; DSM: Diagnostic and Statistical Manual; HERMES: Heart Failure Molecular Epidemiology for Therapeutic Targets; HF: heart failure; ICD, International Classification of Diseases; MI: myocardial infarction; NICM: nonischemic cardiomyopathy; PGC‐ED: Eating Disorders Working Group of the Psychiatric Genomics Consortium.

### 2.2 Phenotype definition

#### 2.2.1 AN phenotypes

Lifetime AN was diagnosed based on three criteria: (1) BMI lower than minimally expected; (2) intense fear of gaining weight; and (3) weight or shape disturbance, undue influence of weight or shape, or denial of the seriousness of the disorder. AN with binge eating included individuals who had lifetime AN and engaged in binge‐eating behavior (i.e., eating a large amount of food in a short period of time while having a sense of loss of control over the eating episode). AN without binge eating included individuals who reported lifetime AN, but no binge‐eating episodes. Although AN subtypes included in the Diagnostic and Statistical Manual were binge‐eating/purging subtype and restricting subtype, we could not examine the differences between these subtypes as they were not captured in the most recent PGC‐ED GWAS (Watson et al., 2019). Thus, we examined the differences by presence of binge‐eating episodes to provide initial information about the role of binge eating in shared genetic risks between AN and CVD.

#### 2.2.2 CVD phenotypes

Detailed descriptions can be found elsewhere (Aragam et al., 2018; Hartiala et al., 2021; Malik et al., 2018; Roselli et al., 2018; Shah et al., 2020; van der Harst & Verweij, 2018). Atrial fibrillation cases were participants with paroxysmal or permanent atrial fibrillation, or atrial flutter. CAD was defined using International Classification of Diseases (ICD)−10 codes, Office of Population Censuses and Surveys Classification of Interventions and Procedures, version 4 codes, and self‐reported CAD. Heart failure cases were participants with a clinical diagnosis of heart failure of any etiology with no inclusion criteria based on left ventricular ejection fraction. Myocardial infarction was defined based on ICD‐10 for myocardial infarction and complications following acute myocardial infarction, doctor‐diagnosis, self‐report, and echocardiographic results. NICM was defined based on left ventricular dysfunction and the absence of CAD among patients with heart failure. Stroke was defined as ischemic stroke or intracerebral hemorrhage based on clinical and imaging criteria.

### 2.3 Statistical analysis

We calculated single nucleotide polymorphisms (SNP)‐based heritability for each phenotype and genetic correlations between each phenotype using linkage disequilibrium score regression (LDSC; Bulik‐Sullivan et al., 2015a, 2015b). Heritability, ranging from 0 to 1, measures the proportion of variation in a phenotype accounted by genetic factors. Genetic correlation, ranging from −1 to +1, measures the extent to which two phenotypes share common genetic variation. Positive and negative genetic correlations indicate that the same genetic factors contribute to variation in two phenotypes in the same and opposite directions, respectively. We used the false discovery rate (FDR; Benjamini et al., 2001) to correct for multiple testing (*n* = 28 tests). Genetic correlations with FDR‐p (*q*) values smaller than .05 were considered significant.

## 3 RESULTS

The SNP‐based heritability was 0.18 (*SE* = 0.01) for AN, 0.21 (*SE* = 0.04) for AN with binge eating, and 0.21 (*SE* = 0.05) for AN without binge eating. For CVD events, the SNP‐based heritability ranged from 0.00 for NICM to 0.13 for CAD. We did not include NICM in the subsequent analyses due to a non‐significant heritability estimate.

**FIGURE 1:**
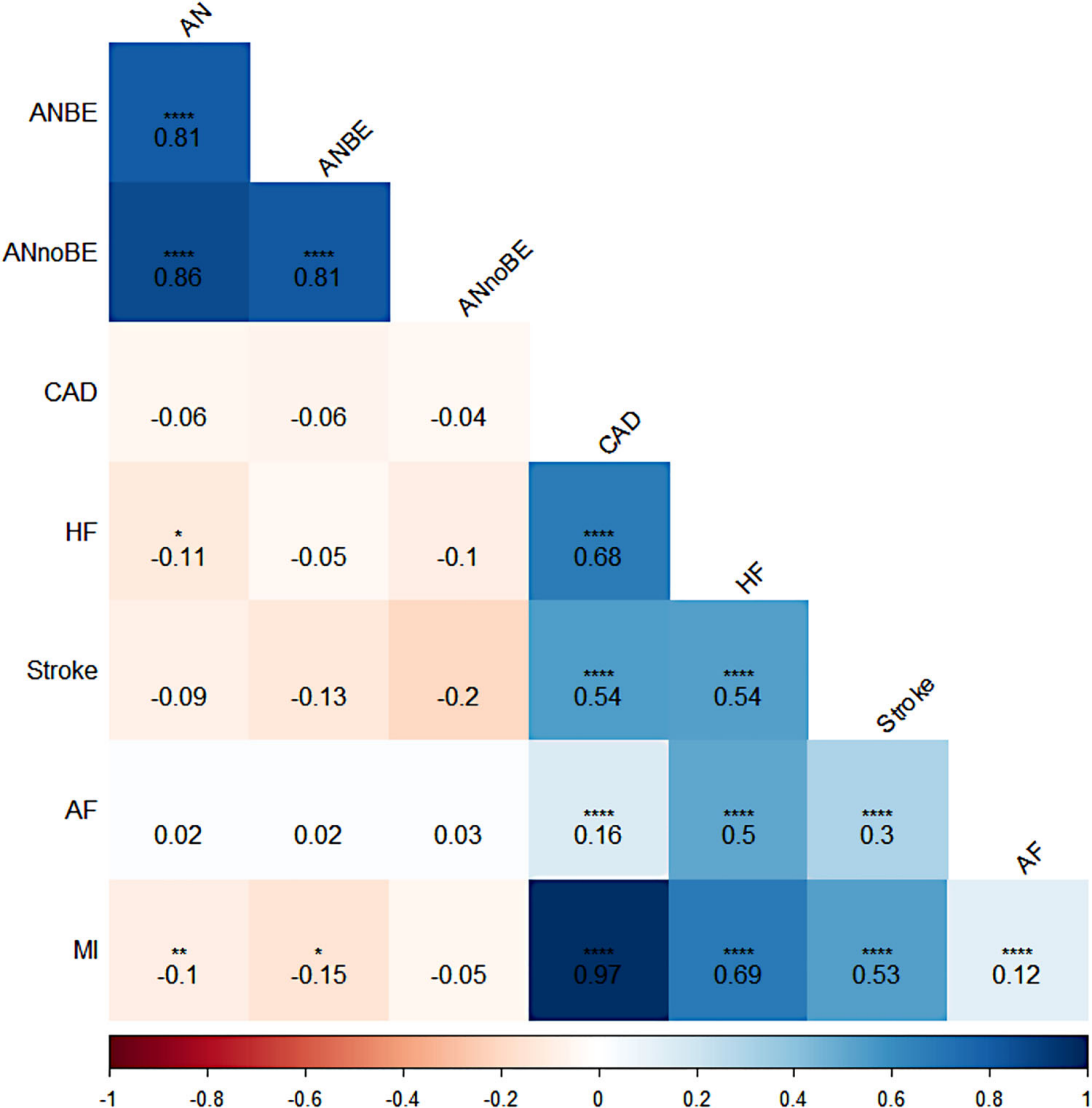
Genetic correlations between anorexia nervosa and cardiovascular disease events. AF: atrial fibrillation; AN: anorexia nervosa; ANBE: anorexia nervosa with binge eating; ANnoBE: anorexia nervosa without binge eating; CAD: coronary artery disease; HF: heart failure; MI: myocardial infarction. Starred values denote significant genetic correlations after correcting for multiple comparisons using False‐Discovery Rate (*n* tests = 28, ****q < .0001, ***q < .001, **q < .01, *q < .05).

Results for genetic correlations between each phenotype are presented in Table S1 and Figure 1. Significant negative genetic correlations emerged between AN phenotypes with CVD events, even though the magnitudes were small. Specifically, AN showed a significant negative genetic correlation with heart failure (*r_g_* = −0.11; 95% confidence interval (CI): −0.21, −0.02; *q* = .04) and myocardial infarction (*r_g_* = −0.10; 95% CI: −0.16, −0.03; *q* = .01). AN with binge eating also showed a significant negative genetic correlation with myocardial infarction (*r_g_* = −0.15; 95% CI: −0.26, −0.04; *q* = .02). No statistically significant genetic correlation was observed between AN without binge eating with any CVD event. All CVD events showed significant positive genetic correlations with each other, with the genetic correlations ranging from 0.12 between atrial fibrillation and myocardial infarction to 0.97 between CAD and myocardial infarction.

## 4 DISCUSSION

Using summary statistics from GWAS, we found negative genetic correlations between AN with heart failure and myocardial infarction, which was consistent with previous findings suggesting negative genetic correlations between AN with CVD risk factors (Duncan et al., 2017; Watson et al., 2019). Genetic loci affect the risk for both AN and CVD, but in the opposite direction. Furthermore, these associations differed somewhat by the presence or absence of binge eating.

Our findings suggest that individuals with a high genetic risk of AN have a low genetic risk for CVD. Clinically, however, cardiovascular events in AN are quite common (Mehler et al., 2022; Sachs et al., 2016), suggesting that the setting of AN itself, characterized by starvation, malnutrition, low body weight, and in some, excessive exercise or purging, may have important direct effects on cardiac functioning and may not be operating through genetics. Many AN‐associated cardiovascular complications have been considered a sequalae of chronic starvation and weight loss, such as reduced left ventricular mass index and reduction in chamber dimensions (Mehler & Andersen, 2017), which are both independent risk factors for sudden cardiac death (Laukkanen et al., 2014; Narayanan et al., 2014). Furthermore, some individuals with AN engage in excessive exercise or purging behaviors, which are associated with adverse cardiovascular effects, including electrolytic disorders and bradycardia (El Ghoch et al., 2013; Giovinazzo et al., 2019). Therefore, our study provides further support for the important role of AN‐associated pathologic changes in high CVD risks.

We also observed differences in the genetic correlation between AN with CVD events by presence of binge eating. AN with binge eating was only genetically correlated with myocardial infarction, and AN without binge eating was not genetically correlated with any CVD event, which contrasted with our hypotheses. These results suggest a possibly distinct pathway of genes in those with AN by the presence of binge eating, yet replication of results is needed in a larger sample. The significant genetic correlation between AN with heart failure did not remain significant in both subtypes, which is probably related to insufficient statistical power in AN subtypes. Future research with larger sample sizes could further clarify potential differences by AN subtype.

Our study provided important evidence on the nature of the association between AN and CVD, yet several limitations must be considered. First, the sample size for each trait varied substantially, ranging from 12,516 for AN without binge eating to 977,323 for heart failure, which likely had important consequences for our study findings. The PGC‐ED is currently working on the next freeze of GWAS, which consists of a larger sample size that could reduce the imbalance of sample sizes. Second, we could not stratify our analyses by sex because most participants in the AN GWAS were female. With the PGC‐ED actively recruiting male participants, further research should investigate how shared genetic risks between AN and CVD differ by sex. Lastly, all samples were primarily of European ancestry; therefore, we could not conduct ancestry‐specific analysis due to limited statistical power. Thus, our findings could be most relevant for European‐ancestry populations. With most consortia currently recruiting more diverse study populations, we hope to investigate ancestral differences in shared genetics of AN and CVD in the future.

In conclusion, our study provides novel evidence regarding shared genetic risks between AN and CVD events. The modest negative correlations of AN with CVD suggest that cardiovascular events seen clinically in individuals with AN are unlikely due to high genetic risk, but rather downstream consequences of AN‐associated pathologic changes secondary to prolonged starvation and weight loss. Future research should elucidate shared genetic factors of AN and CVD by sex and ancestry using larger and more diverse populations, as well as investigate the difference in CVD risks by AN subtype.

## AUTHOR CONTRIBUTIONS

**Baiyu Qi**: Conceptualization; data curation; formal analysis; investigation; software; visualization; writing—original draft. **Mariaelisa Graff**: Conceptualization; writing—review and editing. **Eating Disorders Working Group of the Psychiatric Genomics Consortium**: Resources. **Cynthia M Bulik**: Funding acquisition; writing—review and editing. **Kari E North**: Conceptualization; supervision; writing—review and editing. **Melissa A Munn‐Chernoff**: Conceptualization; funding acquisition; supervision; writing—review and editing.

## CONFLICT OF INTEREST STATEMENT

CMB reports: Shire (grant recipient, Scientific Advisory Board member); Lundbeckfonden (grant recipient); Pearson (author, royalty recipient); Equip Health Inc. (Clinical Advisory Board). All other authors report no conflicts of interest.

### PEER REVIEW

The peer review history for this article is available at https://publons.com/publon/10.1002/brb3.3294.

## Supporting information

Table S1. Linkage disequilibrium score regression results.

## Data Availability

Genome‐wide summary statistics for anorexia nervosa phenotypes are freely downloadable from the website of the PGC (https://pgc.unc.edu/for‐researchers/download‐results/). Genome‐wide summary statistics for other phenotypes can be downloaded from GWAS Catalog at https://www.ebi.ac.uk/gwas/studies/GCST005195 for coronary artery disease, https://www.ebi.ac.uk/gwas/studies/GCST009541 for heart failure, https://www.ebi.ac.uk/gwas/studies/GCST007714 for nonischemic cardiomyopathy, https://www.ebi.ac.uk/gwas/studies/GCST005838 for stroke, https://www.ebi.ac.uk/gwas/studies/GCST006061 for atrial fibrillation, and https://www.ebi.ac.uk/gwas/studies/GCST011365 for myocardial infarction.
